# Screening of *Forsythia suspensa*-derived compounds identifies amentoflavone as a ciprofloxacin-potentiating candidate against MRSA

**DOI:** 10.3389/fmicb.2026.1919329

**Published:** 2026-09-03

**Authors:** Yu Fang, Tianxiang Yue, Jia Zhong, Hangsheng Zheng, Fanzhu Li, Lai Jiang

**Affiliations:** School of Pharmaceutical Sciences, Zhejiang Chinese Medical University, Hangzhou, China

**Keywords:** amentoflavone, antibiotic adjuvant, efflux-associated activity, MRSA, natural product, NorA

## Abstract

Methicillin-resistant *Staphylococcus aureus* (MRSA) remains a clinically important pathogen, and bacterial efflux systems can reduce intracellular antibiotic accumulation and compromise antibacterial efficacy. NorA, a well-studied multidrug efflux pump in *S. aureus*, is associated with the efflux of fluoroquinolones and fluorescent substrates such as ethidium bromide (EtBr), making it a potential target for antibiotic adjuvant discovery. In this study, 100 PubChem-identifiable *Forsythia suspensa (F. suspensa)*-derived small molecules were screened against NorA using CB-Dock2 with AutoDock Vina scoring. Amentoflavone (AME), a naturally occurring biflavonoid, was identified as the top-ranked candidate and displayed a predicted binding pose within the NorA transmembrane cavity, with multiple potential interactions involving residues such as Thr336, Arg310, and Ser133. Molecular dynamics simulation performed using GROMACS 2025.4 further supported the dynamic stability of the predicted AME-NorA complex in a membrane-embedded environment. Functionally, AME increased intracellular EtBr-associated fluorescence in MRSA, suggesting altered EtBr-associated intracellular accumulation or efflux-associated activity. Although AME alone showed no evident direct anti-MRSA activity at concentrations up to 512 μg/mL, its combination with ciprofloxacin enhanced ciprofloxacin-mediated inhibition and killing of MRSA. Specifically, AME reduced the minimum inhibitory concentration (MIC) of ciprofloxacin from 1 to 0.5 μg/mL and the minimum bactericidal concentration (MBC) from 2 to 1 μg/mL. These findings suggest that AME may serve as a *F. suspensa*-derived ciprofloxacin-potentiating candidate against MRSA, potentially associated with altered efflux-related activity. Further studies using NorA genetic models, membrane permeability assays, intracellular ciprofloxacin accumulation assays, and multiple clinical isolates are needed to clarify the target specificity and broader applicability of AME-mediated ciprofloxacin potentiation.

## 1 Introduction

Methicillin-resistant *Staphylococcus aureus* (MRSA) is one of the important drug-resistant pathogens in clinical infections, capable of causing various diseases such as skin and soft tissue infections, pneumonia, bacteremia, bone and joint infections, and infections related to medical devices (Tong et al., 2015; Lee et al., 2018). Due to MRSA carrying methicillin-resistant related resistance mechanisms, it is resistant to most β-lactam antibiotics, and during clinical treatment, it can further acquire resistance to multiple classes of antimicrobial drugs, making its infection treatment still face significant challenges. Although vancomycin, linezolid, and dalbavancin remain important options for treating MRSA infections, issues such as limited choices of antimicrobial agents, increased risk of resistance, and recurrent infections have prompted researchers to continuously explore new anti-infection strategies (Parsons et al., 2026). In this context, using non-antibiotic molecules to enhance the activity of existing antibiotics has become an important strategy for antibiotic adjuvant discovery.

Ciprofloxacin (Cip) belongs to the fluoroquinolone class of antibiotics and exerts its antibacterial effect primarily by inhibiting bacterial DNA gyrase and topoisomerase IV, thereby interfering with the DNA replication process (Hooper and Jacoby, 2016). However, the antibacterial activity of ciprofloxacin in *Staphylococcus aureus* (*S. aureus*) can be affected by various resistance mechanisms, including target enzyme mutations, enhanced drug efflux, and bacterial adaptive tolerance. Among them, the drug efflux mediated by efflux pumps can reduce the effective concentration of antibiotics within bacterial cells, making it one of the important factors affecting the activity of fluoroquinolone antibiotics. NorA is a well-studied multidrug efflux pump in *S. aureus*, belonging to the major facilitator superfamily (MFS) of transport proteins (Papkou et al., 2020; Brawley et al., 2022). It can mediate the efflux of fluoroquinolone drugs such as ciprofloxacin and norfloxacin, as well as structurally unrelated small molecules like ethidium bromide (EtBr). Patel et al. (2010) study on bloodstream *S. aureus* clinical isolates showed that among strains with enhanced efflux phenotypes and increased expression of efflux pump genes, elevated *norA* expression was quite common, suggesting that NorA plays a significant role in efflux-related resistance in clinical isolates.

Modulating NorA-related efflux activity is a feasible strategy to enhance the intracellular accumulation and antibacterial activity of fluoroquinolones. Several reported NorA-related efflux pump inhibitors or efflux-modulating compounds have been shown to reduce the MIC of fluoroquinolones against *S. aureus*, especially in NorA-overexpressing or fluoroquinolone-resistant strains (Mahey et al., 2021; Chandal et al., 2023; Momin-Reza et al., 2025; Cernicchi et al., 2025). However, many reported efflux-modulating compounds still face limitations, including modest activity, insufficient target specificity, potential cytotoxicity, or unclear translational potential. Therefore, continuing to search for small molecules with defined sources and antibiotic-potentiating potential remains of significant research value.

Natural products possess structural diversity and rich biological activity, making them an important source for discovering antibacterial adjuvants candidates. Especially flavonoids, polyphenols, and alkaloids derived from plants have been reported to enhance the activity of antibiotics by affecting bacterial efflux pump activity, membrane permeability, oxidative stress, or biofilm formation (dos Santos Barbosa et al., 2021; Ahmad et al., 2024; Santos et al., 2023; Tintino et al., 2023; Araújo et al., 2025; de Menezes Dantas et al., 2025). *Forsythia suspensa* (*F. suspensa*) is a commonly used medicinal plant that contains various phenylethanoid glycosides, lignans, flavonoids, and biflavonoids (Wang et al., 2018). Its anti-inflammatory, antioxidant, and anti-infection activities have been studied to some extent. Amentoflavone (AME) is a naturally occurring biflavonoid. A previous study reported that AME showed synergistic antibacterial effects with several conventional antibiotics, including ampicillin, cefotaxime, and chloramphenicol, although the underlying mechanisms may vary depending on the bacterial species and antibiotic tested (Hwang et al., 2013). However, whether AME can potentiate ciprofloxacin activity against MRSA through an efflux-related mechanism remains unclear.

Based on this background, this study constructed a candidate compound library containing 100 PubChem-identifiable small molecules derived from *F. suspensa* and performed virtual screening against the *S. aureus* NorA efflux pump. Molecular docking and molecular dynamics (MD) simulation were used to evaluate the structural plausibility and dynamic stability of the selected AME-NorA complex. Subsequently, the effect of AME on efflux-associated activity in MRSA was evaluated using an EtBr accumulation assay, and its ability to enhance ciprofloxacin-mediated anti-MRSA activity was assessed using the minimum inhibitory concentration (MIC)/minimum bactericidal concentration (MBC) determination, checkerboard assay, and spot-plating validation. This study aimed to preliminarily evaluate AME as a *F. suspensa*-derived ciprofloxacin-potentiating candidate and to provide initial evidence for natural-product-derived antibiotic adjuvant discovery.

## 2 Methods

### 2.1 Molecular docking-based virtual screening

A total of 177 *F. suspensa*-derived small molecules were initially retrieved from Traditional Chinese Medicine Systems Pharmacology Database (TCMSP) for virtual screening. After excluding entries without valid PubChem IDs and removing duplicated PubChem records, 100 unique PubChem-identifiable compounds were retained for molecular docking. The corresponding ligand structures were obtained from PubChem in SDF format. The three-dimensional structure of the *S. aureus* NorA efflux pump was obtained from the Protein Data Bank (PDB 9B3K). Water molecules, co-crystallized ligands, and other non-protein heteroatoms were removed before docking.

NorA-IN-1 and Boeravinone B were included as reported NorA-related reference compounds and processed using the same workflow. Blind docking was performed using CB-Dock2, which automatically detects potential binding cavities and generates docking boxes for AutoDock Vina-based docking (Liu et al., 2022; Yang et al., 2022; http://183.56.231.194:8001/cb-dock2/index.php). Therefore, no manually predefined grid box was used in this study. Each compound was docked five times, and the mean Vina score was used for ranking. The compounds were ranked from the most negative to the least negative mean Vina score. AME, the top-ranked compound, was selected for detailed interaction analysis and subsequent molecular dynamics simulation. Three-dimensional binding poses were visualized using PyMOL 3.1.0a0, and two-dimensional interaction diagrams were generated using LigPlot+ 2.2.5.

### 2.2 Molecular dynamics simulation

MD simulation was performed to evaluate the dynamic stability of the predicted AME-NorA complex. The representative docked AME-NorA complex obtained from the molecular docking analysis was used as the initial conformation for MD simulation.

All MD simulations were carried out using GROMACS 2025.4 with the CHARMM36m force field. The AME-NorA complex was placed in a simulation box, and the minimum distance between the solute and the box edge was set to at least 1.0 nm to satisfy periodic boundary conditions. The system was solvated using the TIP3P water model. Counter ions, including Na^+^ or Cl^−^, were added using the gmx genion module to neutralize the total charge of the system and to mimic a physiological salt concentration of 0.15 M. Before production simulation, the system was subjected to energy minimization using the steepest descent algorithm for a maximum of 50,000 steps, with a convergence criterion of maximum force below 1,000 kJ/mol/nm, to remove unfavorable atomic contacts. The minimized system was then equilibrated in two consecutive steps. First, an NVT equilibration was performed for 100 ps at 310.15 K using the V-rescale thermostat with a coupling constant of 0.1 ps. Subsequently, an NPT equilibration was conducted for 100 ps at 1 bar using the Parrinello-Rahman barostat with a coupling constant of 2.0 ps. After equilibration, an unrestrained production MD simulation was performed for 100 ns under the NPT ensemble. The integration time step was set to 2 fs, and trajectory coordinates were saved every 10 ps.

During the simulation, van der Waals and short-range electrostatic interactions were treated with a cutoff distance of 1.2 nm. Long-range electrostatic interactions were calculated using the particle mesh Ewald (PME) method, and all bonds involving hydrogen atoms were constrained using the LINCS algorithm. After simulation, the trajectories were analyzed using built-in GROMACS tools. Root mean square deviation (RMSD) was calculated to evaluate the structural stability and equilibration of the complex. The radius of gyration (Rg) was analyzed to assess the compactness of the AME-NorA complex. Solvent-accessible surface area (SASA) was calculated to monitor changes in solvent exposure during the simulation. Hydrogen bond analysis was performed to evaluate the dynamic intermolecular interactions between AME and NorA. Root mean square fluctuation (RMSF) was used to assess residue-level flexibility of NorA during simulation. In addition, the free energy landscape (FEL) was constructed using RMSD and Rg as reaction coordinates to evaluate the conformational distribution and low-energy states of the AME-NorA complex. To further estimate the binding energetics of the AME-NorA complex, MM/PBSA binding free energy analysis was performed using frames extracted from the stable stage of the MD trajectory. Considering that the ligand RMSD reached a relatively stable state after approximately 30 ns, the trajectory from 30 to 100 ns was used for MM/PBSA calculation. The receptor and ligand were defined as NorA and AME, respectively. Binding free energy components, including van der Waals, electrostatic, polar solvation, and nonpolar solvation terms, were calculated. Per-residue energy decomposition was further performed to identify residues contributing to AME stabilization within the NorA binding cavity. The entropic contribution was not included in the final binding free energy estimation.

Membrane-normal binding depth was calculated after transforming the NorA-AME complex into the PDBTM membrane coordinate frame. In this frame, the membrane normal is parallel to the *Z* axis, the membrane midplane is located at *Z* = 0, and the predicted hydrophobic boundaries are located at *Z* = ±15.25 Å. The negative-Z face was assigned as extracellular based on the position of the extracellularly bound Fab36 in the experimental 9B3K complex. The binding-site center was operationally defined as the geometric centroid of the AME heavy atoms, and depth was measured along the Z axis from the extracellular hydrophobic boundary. As a complementary phospholipid-headgroup reference, a static POPG model was used to define extracellular and cytoplasmic phosphorus median planes.

### 2.3 Chemicals and bacterial strain

AME was commercially purchased from MedChemExpress (Catalog No. HY-N0662; MCE, Shanghai, China) with a purity of ≥99.66%, as stated by the supplier. AME was dissolved in dimethyl sulfoxide (DMSO) to prepare a 1 mM stock solution and stored at −20 °C until use. Ciprofloxacin was purchased from MedChemExpress (Catalog No. HY-B0356) and prepared according to the manufacturer's instructions. For all antibacterial experiments, working solutions were freshly diluted in the corresponding assay medium.

The MRSA strain used in this study was USA300, which is a laboratory reference strain. The strain was obtained from BNCC (Beijing, China). The strain was maintained on tryptic soy agar (TSA) plates and cultured in tryptic soy broth (TSB) at 37 °C before experiments.

### 2.4 EtBr accumulation assay

The effect of AME on efflux-associated activity in MRSA was evaluated using an EtBr accumulation assay. Briefly, MRSA was cultured overnight in TSB at 37 °C with shaking. The overnight culture was diluted into fresh TSB and grown to the logarithmic phase. Bacterial cells were then harvested by centrifugation, washed twice with phosphate-buffered saline (PBS), and resuspended in PBS or assay buffer to an optical density of OD600 = 0.05. EtBr was added to the bacterial suspension at a final concentration of 1 μg/mL, and the suspension was transferred into a black 96-well microplate.

Fluorescence intensity was monitored using a microplate reader with excitation and emission wavelengths set at 525 and 600 nm, respectively. Baseline fluorescence was recorded for 30 min before AME and Reserpine treatment. AME or Reserpine was then added to the indicated wells at a final concentration of 20 μM, while control wells received the same volume of vehicle. Fluorescence signals were continuously recorded at 10 min intervals for 70 min at 37 °C. The MRSA + EtBr group was used to evaluate basal EtBr accumulation, the MRSA + EtBr + Reserpine group was used as a pharmacological reference control, and the MRSA + EtBr + AME group was used to assess the effect of AME on intracellular EtBr accumulation. All experiments were performed in at least three independent replicates.

### 2.5 Broth microdilution and checkerboard antibacterial assay

The antibacterial activities of ciprofloxacin and amentoflavone, alone or in combination, were evaluated against MRSA using a broth microdilution-based assay. Briefly, MRSA was cultured overnight in TSB at 37 °C with shaking. The overnight culture was diluted into fresh medium and grown to the logarithmic phase. The bacterial suspension was adjusted to approximately 10^6^ CFU/mL and used as the inoculum for the antibacterial assay.

For single-agent testing, two-fold serial dilutions of ciprofloxacin and AME were prepared in a sterile 96-well microplate. Ciprofloxacin was tested over a concentration range of 0.0625–16 μg/mL, and AME was tested over a concentration range of 2–512 μg/mL. Each well contained bacterial suspension and the indicated concentration of compound in a final volume of 100 μL. Wells containing bacteria without treatment were used as growth controls, and wells containing medium only were used as sterility controls. After incubation at 37 °C for 18 h, bacterial growth was evaluated by measuring OD600 or by visual inspection. The MIC was defined as the lowest concentration that completely inhibited visible bacterial growth.

To evaluate the combined antibacterial effect of ciprofloxacin and AME, a checkerboard assay was performed in 96-well plates. Two-fold serial dilutions of ciprofloxacin were prepared along the horizontal axis, while two-fold serial dilutions of AME were prepared along the vertical axis. MRSA suspension was then added to each well to obtain a final bacterial density of approximately 10^6^ CFU/mL. The plates were incubated at 37 °C for 18 h. Bacterial viability under each concentration combination was assessed by OD600 measurement or visual growth evaluation and presented as a concentration-response matrix. The combined effect was evaluated based on the shift in ciprofloxacin MIC in the presence of AME compared with ciprofloxacin alone.

For MBC determination and spot-plating validation, aliquots from selected wells showing no visible growth or representative concentration combinations were collected and spotted onto TSA plates. After incubation at 37 °C for 24 h, colony formation was recorded. The MBC was defined as the lowest drug concentration that resulted in no visible colony growth on agar plates. Representative ciprofloxacin-AME combinations were further compared with ciprofloxacin alone to validate the potentiation of ciprofloxacin-mediated bacterial killing by AME. All experiments were performed in at least three independent replicates. Because AME did not reach a clearly defined MIC within the tested concentration range, the combined antibacterial effect was interpreted based on the reduction of ciprofloxacin MIC/MBC and spot-plating validation rather than standard FICI calculation.

### 2.6 Statistical analysis

All experiments were performed in at least three independent biological replicates unless otherwise stated. Data are presented as mean ± standard deviation (SD). Statistical analyses were performed using GraphPad Prism 10.4.2. For comparisons among multiple groups, one-way ANOVA followed by Tukey's *post hoc* test was used where appropriate. A value of *p* < 0.05 was considered statistically significant.

## 3 Results

### 3.1 Virtual screening identified amentoflavone as a top-ranked *Forsythia suspensa*-derived candidate with NorA-interacting potential

**Figure 1:**
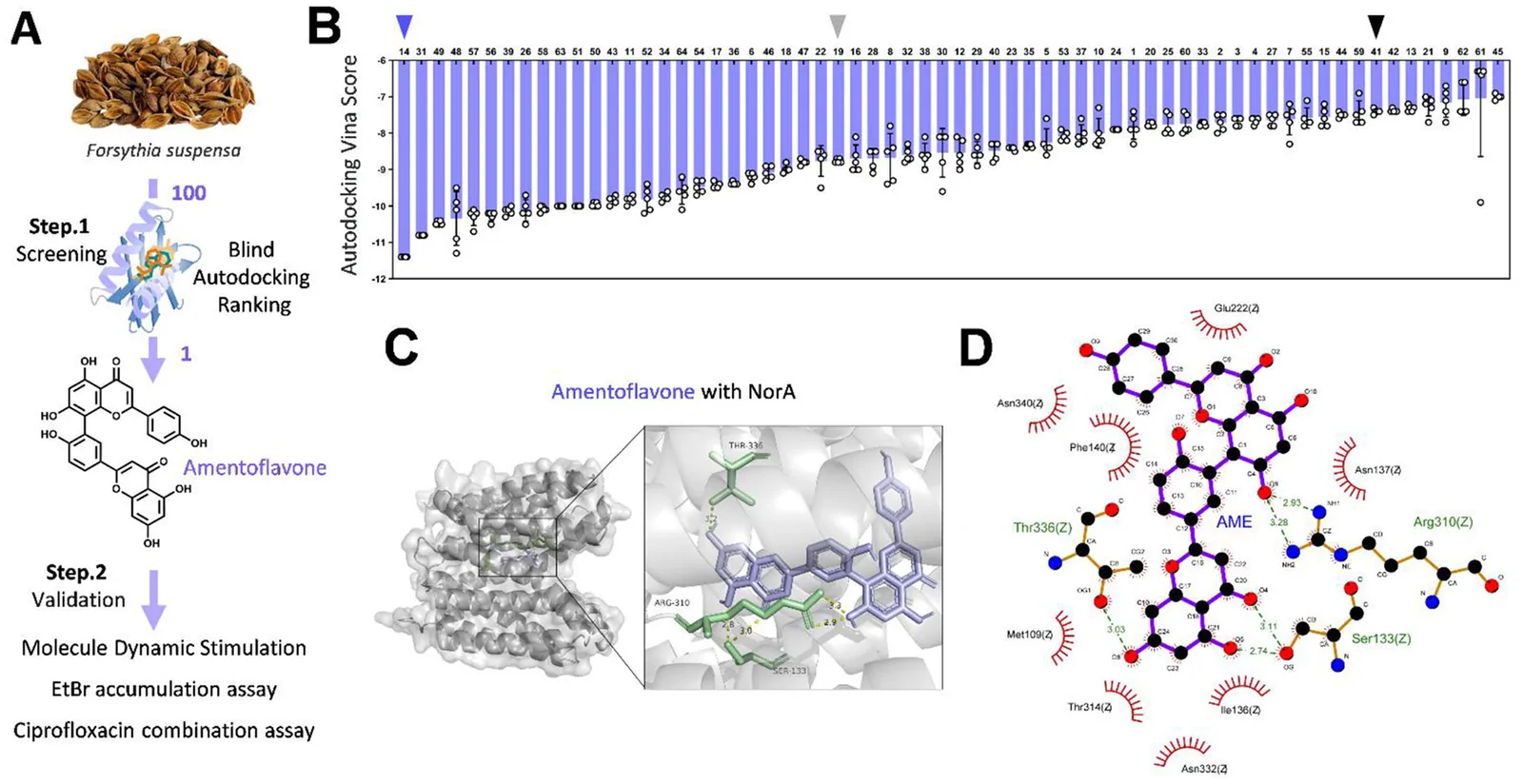
Identification of amentoflavone as a candidate NorA-interacting compound from *F. suspensa*. **(A)** Workflow for virtual screening and experimental validation. In total, 100 PubChem-identifiable compounds derived from *F. suspensa* were screened by blind docking against the NorA efflux pump, followed by candidate selection and validation through molecular dynamics simulation, EtBr accumulation assay, and ciprofloxacin combination assay. Created in BioRender. Jiang, L. (2026) https://BioRender.com/n0dcv0l. **(B)** AutoDock Vina score ranking of the 100 candidate compounds. AME showed the most favorable predicted Vina score and was selected for further analysis, as indicated by the blue arrowhead. The black arrowhead indicates the reference compound NorA-IN-1, and the gray arrowhead indicates the reference compound Boeravinone B. **(C)** Three-dimensional docking model showing the predicted binding pose of AME in NorA. Key residues surrounding the binding region are highlighted in the enlarged panel. **(D)** Two-dimensional interaction map of the AME-NorA complex, indicating multiple potential interactions between amentoflavone and residues within the predicted binding region.

The overall screening strategy of this study is shown in Figure 1A. The study first constructed a candidate compound library containing 100 small molecules derived from Forsythia, and performed blind docking screening using the *S. aureus* NorA structure as the docking receptor. Subsequently, based on the predicted docking scores, the candidate molecules were ranked, and the top-ranked molecule was further evaluated by molecular dynamics simulation, EtBr accumulation assay, and ciprofloxacin combination assays.

The AutoDock Vina docking results showed clear differences in the predicted NorA-binding potential among the screened compounds (Figure 1B and Supplementary Table 1). Across the retained compound library, the mean Vina scores ranged from −11.4 to −4.36 kcal/mol. AME showed the most negative mean Vina score of −11.4 kcal/mol and was therefore ranked as the top candidate in this screening. For comparison, the reference NorA-related compounds NorA-IN-1 and Boeravinone B showed mean Vina scores of −7.4 and −8.76 kcal/mol, respectively. Because a more negative Vina score indicates a more favorable predicted binding mode in this docking system, AME was selected for further structural analysis, MD simulation, and functional validation. The complete docking dataset, including compound names, PubChem IDs, five independent docking scores, mean Vina scores, and ranking information, is provided in Supplementary Table 1.

The three-dimensional binding model of AME with NorA is shown in Figure 1C. AME was predicted to be accommodated within the central binding cavity formed by the transmembrane helices of NorA. To improve consistency between the three-dimensional binding model and the two-dimensional interaction map, the residues highlighted in Figure 1C were selected according to the interaction residues shown in the LigPlot+ analysis in Figure 1D. The 2D interaction diagram showed a multi-site interaction pattern between AME and NorA, involving Thr336, Arg310, Ser133, Glu222, Asn332, Asn340, Phe140, Met109, Thr314, Ile136, and Asn137. Among these residues, Thr336, Arg310, and Ser133 were predicted to form hydrogen bonds or polar interactions with AME, whereas residues such as Phe140, Ile136, Met109, Asn332, and Asn137 were located close to AME and may contribute to local pocket accommodation or weak noncovalent contacts, including van der Waals contacts. As a biflavonoid molecule, AME contains multiple phenolic hydroxyl groups and an extended aromatic framework, which may allow it to adapt to both polar and relatively hydrophobic microenvironments within the NorA binding cavity.

To further evaluate the structural plausibility of the AME docking result, we analyzed the binding modes of the reference NorA-related compounds NorA-IN-1 (Holler et al., 2012a) and Boeravinone B (Singh et al., 2017) under the same docking workflow (Supplementary Figure 1). Both reference compounds were predicted to occupy a binding region within the NorA transmembrane cavity and shared several binding-pocket residues with AME, including Arg310, Ser133, Thr336, Glu222, Asn340, Phe140, Ile136, and Asn137. This partial overlap in binding location and surrounding residues supports the structural plausibility of the predicted AME-NorA interaction. However, these docking results should be interpreted as computational predictions of NorA-interacting potential rather than direct evidence of NorA-specific inhibition.

Together, the blind docking-based screening identified AME as the top-ranked *F. suspensa*-derived candidate among the 100 PubChem-identifiable compounds. AME displayed a favorable predicted binding pose within the NorA transmembrane cavity and shared several binding-pocket residues with reported NorA-related reference compounds. These results provided the basis for subsequent MD simulation and experimental evaluation of AME-associated effects on EtBr accumulation and ciprofloxacin activity against MRSA.

### 3.2 Molecular dynamics simulation supports the dynamic stability and energetic favorability of the AME-NorA complex in a membrane-embedded environment

**Figure 2:**
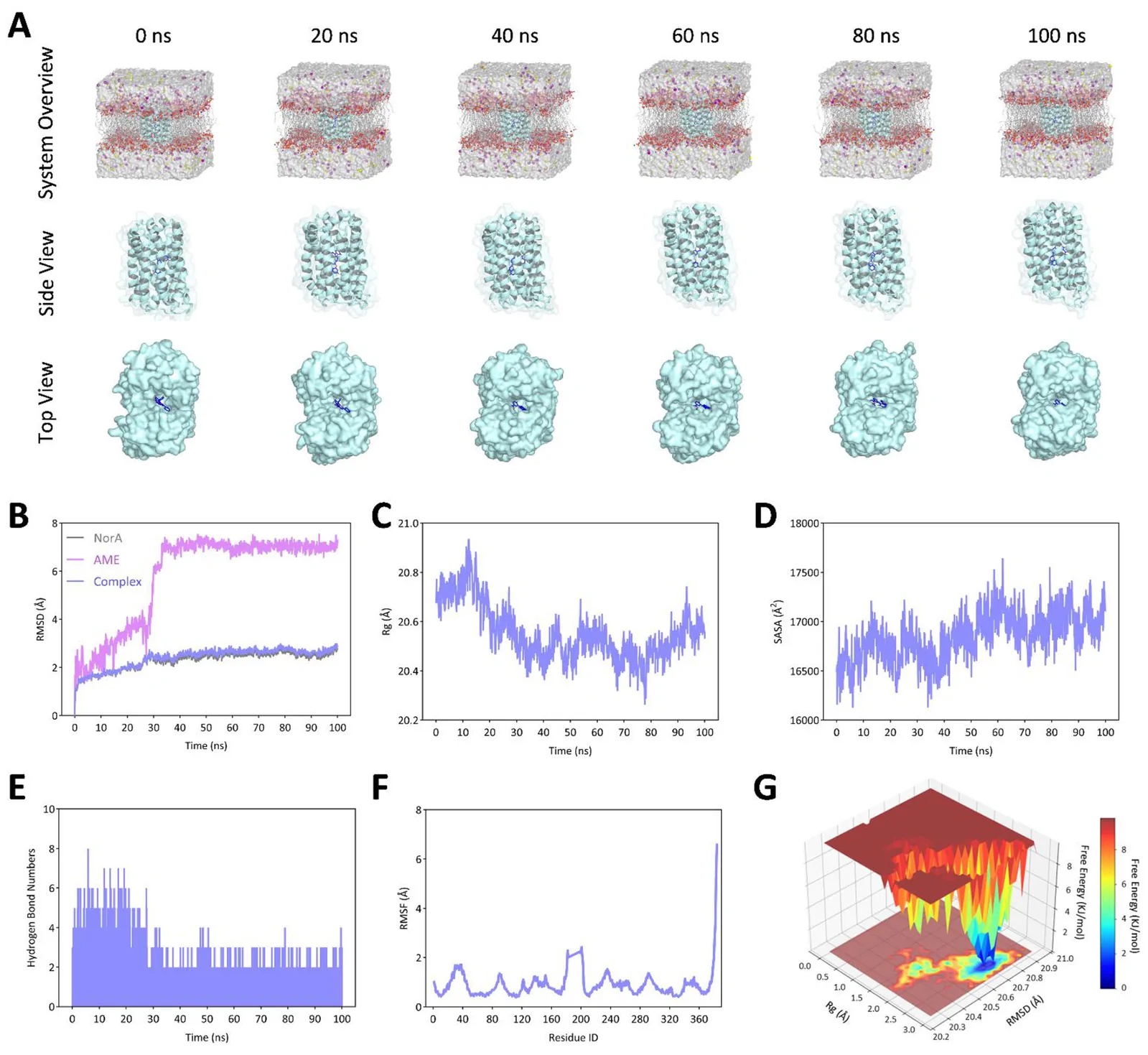
Molecular dynamics simulation of the AME-NorA complex in a membrane-embedded system. **(A)** Representative snapshot of the AME-NorA complex during the 100 ns molecular dynamics simulation at 0, 20, 40, 60, 80, and 100 ns. The complex was analyzed in a membrane-containing environment, and the conformational changes were visualized from system overview, side view, and top view. **(B)** RMSD profiles of NorA, AME, and the AME-NorA complex during the 100 ns simulation. **(C)** Rg profile of the AME-NorA complex, reflecting the overall compactness of the protein-ligand system. **(D)** SASA profile of the AME-NorA complex during the simulation. **(E)** Time-dependent hydrogen bond numbers between AME and NorA, showing the dynamic interaction pattern of the complex. **(F)** RMSF analysis of NorA residues in the AME-NorA complex, indicating residue-level flexibility during the simulation. **(G)** Free energy landscape of the AME-NorA complex constructed based on RMSD and Rg, illustrating the conformational distribution and low-energy states sampled during the simulation.

To evaluate the dynamic stability of the AME-NorA binding model predicted by molecular docking, a 100 ns MD simulation was performed in a membrane-embedded environment. Representative conformational snapshots at different time points are shown in Figure 2A. During the simulation, NorA maintained an intact transmembrane helical bundle structure, without obvious protein unfolding or disruption of the transmembrane region. AME remained generally accommodated within the predicted binding cavity of NorA throughout the simulation, suggesting that the docked binding pose could be maintained under dynamic conditions.

To further define the membrane-relative location of the predicted AME-binding site, a membrane-normal depth analysis was performed using the retained NorA-AME complex. The complex was transformed into the PDBTM membrane coordinate frame, in which the membrane midplane is located at Z = 0 and the predicted hydrophobic boundaries are located at Z = ±15.25 Å (Supplementary Table 3). The negative-Z side was assigned as the extracellular side based on the position of Fab36 in the experimental 9B3K structure (Xie et al., 2025). The AME heavy-atom centroid was located at Z = −2.966 Å, indicating a slight shift toward the extracellular side relative to the membrane midplane (Supplementary Table 4). This corresponded to an inward depth of 12.284 Å from the extracellular PDBTM hydrophobic boundary (Supplementary Figures 4, 5). All AME heavy atoms were located within the predicted membrane slab and spanned 6.912–20.098 Å inward from the extracellular hydrophobic boundary (Supplementary Table 5). Using a complementary static POPG headgroup reference, the extracellular and cytoplasmic phosphorus median planes were located at z = −20.108 and +20.407 Å, respectively (Supplementary Table 3). The AME-centroid depth from the extracellular phosphorus plane was 17.142 Å (Supplementary Figures 4, 5). These results indicate that AME is deeply accommodated within the NorA transmembrane core rather than positioned at a superficial aqueous-exposed region.

RMSD analysis was used to evaluate the structural convergence of the system (Figure 2B). The RMSD of the NorA protein and the AME-NorA complex rapidly increased at the beginning of the simulation and then gradually plateaued, with both showing a similar trend, suggesting that the binding of AME did not cause significant perturbations to the overall conformation of the protein. In contrast, the RMSD of the AME ligand showed a significant increase during the initial 30 ns, after which it maintained a relatively stable plateau state. This indicates that AME underwent an initial conformational adjustment within the binding cavity before reaching a more stable binding state.

Rg analysis further evaluates the structural compactness of the complex (Figure 2C). The Rg value slightly decreased from approximately 20.8 Å to around 20.6 Å at the beginning of the simulation and then fluctuated narrowly around this value in subsequent simulations. This change suggests that the complex underwent moderate conformational contraction during the initial equilibrium phase, while the subsequent stable Rg profile indicates that NorA did not undergo abnormal loosening or expansion after AME binding.

SASA analysis is used to monitor changes in the solvent exposure of the complex (Figure 2D). In the 100 ns simulation, the SASA values fluctuated within the range of 16,000–17,500 Å^2^ and showed a slow upward trend overall. Corresponding to the ligand conformational adjustment around 30 ns in the RMSD, SASA exhibits some fluctuations in the later stages, suggesting that the local microenvironment around the AME binding cavity may undergo rearrangement, causing some previously buried residue surface areas to be re-exposed to the solvent. Importantly, no abrupt or continuous increase in SASA was observed, suggesting that the observed changes were more likely associated with normal structural fluctuation in the membrane-embedded system rather than global destabilization of the complex.

The time-dependent hydrogen bond analysis further revealed the dynamic interaction pattern between AME and NorA (Figure 2E). In the early stages of the simulation, 4–8 hydrogen bonds are formed between AME and NorA, indicating that the ligand can anchor in the binding cavity through multipoint polar contacts. After approximately 30 ns, the number of hydrogen bonds decreases and stabilizes at 2–4. This trend was consistent with the ligand RMSD profile, suggesting that some transient interactions were reorganized during the initial adjustment phase, while the remaining hydrogen bonds may contribute to maintaining the relatively stable AME-binding conformation.

RMSF analysis was used to evaluate residue-level flexibility of NorA during the simulation (Figure 2F). The results show that the RMSF values of the vast majority of residues are below 2 Å, with the transmembrane helix core region being approximately 1 Å. Higher fluctuation peaks were mainly observed at terminal regions and flexible loops, which is consistent with the general dynamic behavior of membrane proteins. This result indicates that the binding of AME does not significantly disrupt the stability of the NorA core structure.

Finally, this study constructed the FEL of the complex based on RMSD and Rg (Figure 2G) to evaluate its conformational distribution characteristics from an energetic perspective. The results show that the system formed a distinct low free energy basin during the simulation process, primarily concentrated around RMSD of approximately 2.7 Å and Rg of approximately 20.7 Å. This low-energy region was consistent with the RMSD, Rg, and hydrogen bond analyses, supporting that the AME-NorA complex reached a relatively stable conformational state after initial structural adjustment. Therefore, the MD results support the dynamic stability of the predicted AME-NorA complex, but should not be overinterpreted as definitive evidence of direct NorA-specific inhibition.

To further provide quantitative energetic support for the predicted AME-NorA interaction, MM/PBSA binding free energy analysis was performed based on the stable-stage MD trajectory (Supplementary Figure 2). The estimated total binding free energy of the AME-NorA complex was −26.53 kcal/mol, indicating that the predicted interaction was energetically favorable. Energy component analysis showed that van der Waals interaction made a major favorable contribution to complex stabilization (ΔE_vdW_ = −53.50 kcal/mol), together with favorable electrostatic interaction (ΔE_elec_ = −34.16 kcal/mol) and nonpolar solvation energy (ΔG_nonpol_ = −5.38 kcal/mol). In contrast, the polar solvation term was unfavorable (ΔG_pol_ = +66.51 kcal/mol), partially offsetting the favorable molecular mechanics contributions.

Per-residue energy decomposition further showed that the favorable residue-level contributions were mainly derived from residues forming the hydrophobic binding environment around AME, including Ile136, Met109, Phe140, Met132, and Ile19. These residues contributed total energy values (ΔG_total_) of −1.9, −1.8, −1.6, −0.9, and −0.6 kcal/mol, respectively. By contrast, several polar residues located near AME, such as Arg310, Glu222, Ser133, Asn340, and Asn137, showed limited or unfavorable total energy contributions, likely due to the offsetting effect of polar solvation penalties. Together, these results suggest that the predicted AME-NorA interaction is energetically favorable and mainly stabilized by van der Waals and nonpolar contacts within the membrane-embedded binding environment.

In summary, the 100 ns molecular dynamics simulation indicates that the AME-NorA complex exhibits overall dynamic stability in a membrane-embedded environment. After approximately 30 ns of conformational adjustment, AME tends to stabilize and is overall positioned within the NorA binding cavity; the overall structure of NorA remains compact, with no significant unfolding or abnormal fluctuations observed. The MM/PBSA and per-residue decomposition analyses further support the energetic favorability of the predicted AME-NorA interaction. These computational results provide a structural and energetic basis for subsequent evaluation of AME-associated effects on EtBr accumulation and ciprofloxacin activity against MRSA.

### 3.3 AME potentiates ciprofloxacin-mediated inhibition and killing of MRSA

Based on the computational results suggesting that AME has NorA-interacting potential, we next evaluated whether AME could affect efflux-associated activity in MRSA using an EtBr accumulation assay. EtBr is a fluorescent substrate commonly used to assess bacterial efflux-related activity, although it is not specific to NorA. Therefore, this assay was used to evaluate whether AME altered EtBr-associated intracellular fluorescence rather than to directly prove NorA-specific inhibition.

**Figure 3:**
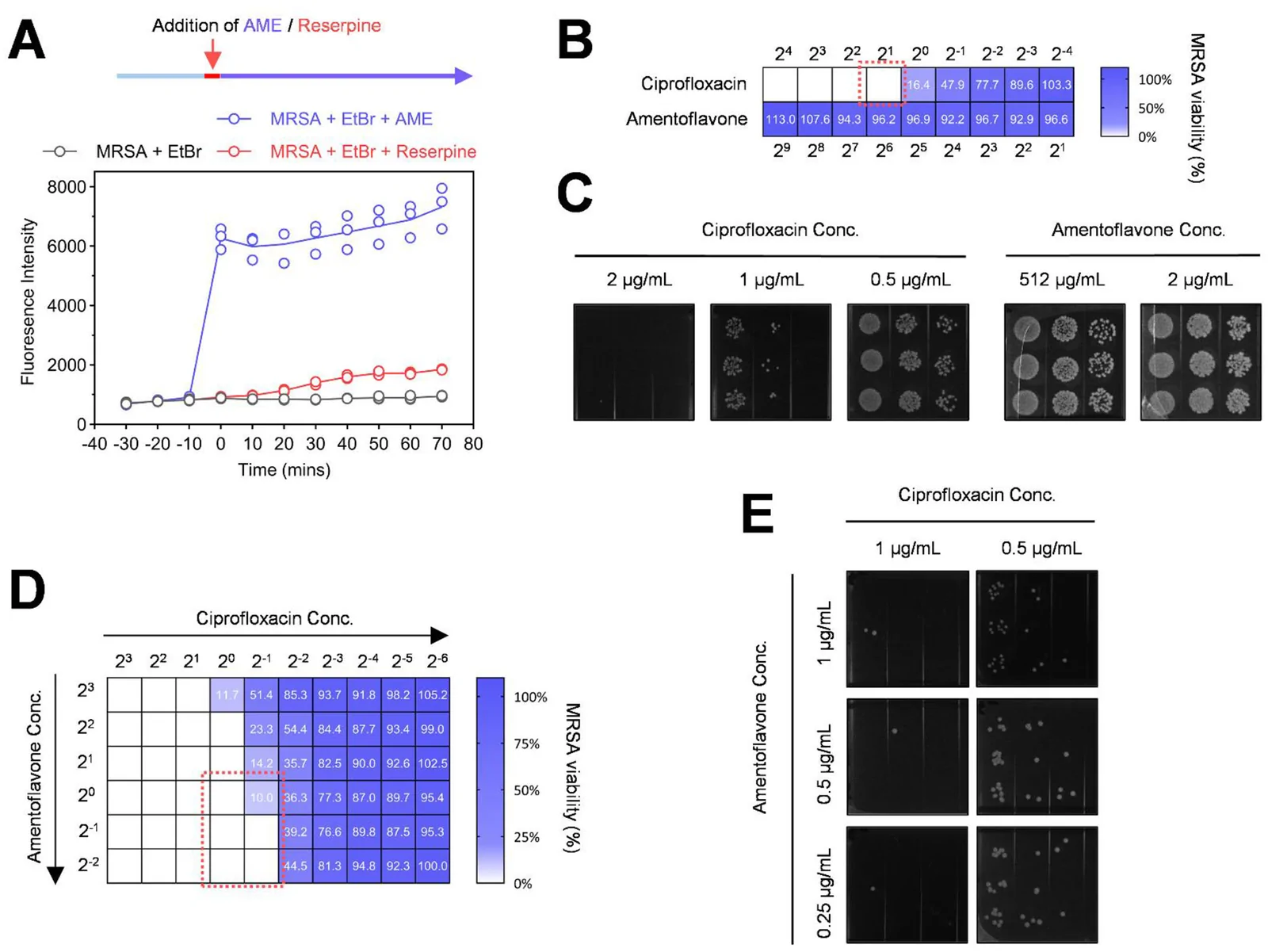
Amentoflavone increases EtBr-associated intracellular fluorescence and potentiates ciprofloxacin-mediated inhibition of MRSA. **(A)** EtBr accumulation assay in MRSA. EtBr-loaded MRSA cells were monitored for fluorescence intensity over time, and AME or Reserpine was added at the indicated time point. **(B)** Broth microdilution assay showing the antibacterial activities of ciprofloxacin and AME alone against MRSA. Bacterial viability is presented as a heatmap, with white indicating complete growth inhibition and blue indicating bacterial survival. The red dashed box indicates the MIC of free ciprofloxacin. **(C)** Spot-plating validation of the antibacterial activities of ciprofloxacin and AME alone at the indicated concentrations. **(D)** Checkerboard assay evaluating the combined antibacterial effect of ciprofloxacin and AME against MRSA. Bacterial viability under different concentration combinations is shown as a heatmap. The red dashed box indicates the concentration combinations showing enhanced ciprofloxacin-mediated inhibition. **(E)** Spot-plating validation of the ciprofloxacin-AME combination.

As shown in Figure 3A, the fluorescence intensity of the MRSA + EtBr control group remained at a relatively low level with only slight fluctuations throughout the entire detection period, indicating limited intracellular EtBr accumulation under basal conditions. Reserpine, a commonly used bacterial efflux inhibitor, was included as a reference control. After reserpine treatment, EtBr-associated fluorescence gradually increased compared with the EtBr-only control, indicating that this assay could detect pharmacologically induced changes in EtBr accumulation. After the addition of AME, the fluorescence intensity in the MRSA + EtBr + AME group rapidly increased and remained at a high level. These results suggest that AME altered EtBr-associated intracellular accumulation in MRSA and are consistent with an efflux-associated interpretation. However, this assay alone does not establish NorA-specific inhibition.

Subsequently, this study separately determined the antibacterial and bactericidal activities of ciprofloxacin and AME against MRSA. As shown in Figure 3B, ciprofloxacin exhibits a clear concentration-dependent antibacterial effect. Quantitative viability analysis showed that ciprofloxacin reduced MRSA viability to −4.22 ± 8.21%, 0.00 ± 6.75%, and 17.57 ± 7.92% at 4, 2, and 1 μg/mL, respectively (Supplementary Table 2). Slight negative values after normalization reflect background correction and were interpreted as near-complete growth inhibition. Based on the broth microdilution results and visual growth assessment, the MIC of ciprofloxacin was determined to be 1 μg/mL. Spot-plating validation further showed that 2 μg/mL ciprofloxacin almost completely eliminated visible colony formation, corresponding to an MBC of 2 μg/mL (Figure 3C). When the concentration of ciprofloxacin was reduced to 0.5 μg/mL, a large number of colonies were still observed, indicating that this concentration was insufficient to completely inhibit the growth of MRSA. In contrast, AME did not exhibit significant direct antibacterial activity within the tested concentration range (up to 512 μg/mL), and the survival rate of MRSA remained consistently high (Figures 3B, C, and Supplementary Figure 3). These results indicate that AME itself is not a potent direct anti-MRSA agent under the present assay conditions.

We further examined whether AME could enhance ciprofloxacin-mediated inhibition of MRSA using a checkerboard concentration matrix. As shown in Figure 3D, the addition of AME shifted the ciprofloxacin inhibition profile toward lower ciprofloxacin concentrations. In the selected AME-containing concentration region, 1 μg/mL ciprofloxacin reduced MRSA viability to −1.54 ± 2.48%, −4.80 ± 6.11%, and 0.00 ± 3.34% in the presence of 1, 0.5, and 0.25 μg/mL AME, respectively (Figure 3D and Supplementary Table 2). More importantly, when ciprofloxacin was reduced to 0.5 μg/mL, the addition of 1, 0.5, and 0.25 μg/mL AME reduced bacterial viability to 10.63 ± 3.92%, 6.57 ± 7.46%, and 3.94 ± 7.96%, respectively. This expansion of the low-viability region toward 0.5 μg/mL ciprofloxacin supports the observation that AME enhanced ciprofloxacin-mediated growth inhibition and reduced the apparent MIC of ciprofloxacin from 1 to 0.5 μg/mL. The three independent repeated datasets corresponding to the single-agent and checkerboard assays are provided in Supplementary Figure 3. Spot-plating validation further supported the potentiating effect of AME on ciprofloxacin-mediated bacterial killing (Figure 3E). Compared with ciprofloxacin alone, the combination treatment reduced visible colony formation at 0.5 μg/mL ciprofloxacin to varying degrees, while the 1 μg/mL ciprofloxacin combination group showed almost no visible colony formation. Based on the spot-plating results, the MBC of ciprofloxacin decreased from 2 to 1 μg/mL when combined with AME, indicating that AME enhanced ciprofloxacin-mediated bactericidal activity against MRSA.

Taken together, AME alone showed limited direct anti-MRSA activity under the tested conditions, but increased EtBr-associated intracellular fluorescence and enhanced ciprofloxacin-mediated inhibition and killing of MRSA. Specifically, AME reduced the MIC of ciprofloxacin from 1 to 0.5 μg/mL and the MBC from 2 to 1 μg/mL. Because AME alone did not reach a clearly defined MIC within the tested concentration range, a conventional fractional inhibitory concentration index (FICI) value was not calculated, and the combination effect was interpreted based on MIC/MBC reduction, checkerboard viability matrix, and spot-plating validation rather than as a formal FICI-defined synergy conclusion. These functional results support AME as a ciprofloxacin-potentiating candidate with possible efflux-associated activity, while further NorA-specific and membrane permeability studies are needed to clarify the precise mechanism.

## 4 Discussion

This study screened *Forsythia suspensa*-derived small molecules for potential antibiotic adjuvant activity against MRSA. AME was identified as the top-ranked candidate among 100 PubChem-identifiable compounds. In the molecular docking analysis, AME showed the most favorable predicted Vina score and was accommodated within the transmembrane cavity of NorA. The subsequent 100 ns molecular dynamics simulation further showed that the AME-NorA complex maintained a relatively stable conformation in a membrane-embedded environment. RMSD, Rg, SASA, hydrogen bond, RMSF, and FEL analyses all supported the dynamic stability of AME within the NorA binding cavity. In addition, MM/PBSA analysis showed a negative estimated binding free energy for the AME-NorA complex. Per-residue energy decomposition suggested that favorable contributions mainly came from residues forming a hydrophobic binding environment, including Ile136, Met109, Phe140, Met132, and Ile19. These results suggest that AME stabilization within NorA may depend largely on van der Waals and nonpolar contacts, rather than only on a few polar hydrogen bonds.

It should be emphasized that molecular docking, MD simulation, and MM/PBSA analysis are computational predictions. They cannot independently prove that AME is a target-confirmed NorA inhibitor. Therefore, these computational results should be interpreted as structural and energetic support for a possible AME-NorA interaction, rather than as direct target-validation evidence. NorA is a transmembrane transporter, and ligand binding may be influenced by the membrane environment, protein conformational dynamics, and local lipid interactions. The MD simulation in this study was designed to evaluate the dynamic stability of the pre-docked AME-NorA complex in a membrane-embedded environment. It was not designed to model AME entry, exit, or directional translocation through NorA. Thus, the predicted binding of AME within the NorA transmembrane cavity should be viewed as a membrane-embedded protein-ligand interaction, not as an isolated protein-ligand event in aqueous solution.

This point is particularly relevant to the structural features of AME. AME is a biflavonoid with an extended aromatic phenolic framework and multiple hydroxyl groups. It therefore has amphiphilic structural features. In the membrane-embedded NorA model, AME was located within the transmembrane cavity and close to the hydrophobic lipid-core region. The membrane-normal depth analysis further showed that the AME heavy-atom centroid was located at z = −2.966 Å relative to the membrane midplane, corresponding to an inward depth of 12.284 Å from the extracellular hydrophobic boundary and 17.142 Å from the extracellular POPG phosphorus plane. All AME heavy atoms were located within the predicted membrane slab. These results support that AME is deeply accommodated within the NorA transmembrane core rather than positioned at a superficial aqueous-exposed region.

Thus, in addition to interactions with NorA pocket residues, the aromatic scaffold of AME may form hydrophobic or van der Waals contacts with nearby lipid acyl chains or hydrophobic residues. Its hydroxyl groups may also contribute to polar contacts with surrounding residues or lipid headgroup-associated regions. This interpretation is consistent with the major favorable van der Waals contribution observed in the MM/PBSA analysis. However, AME-lipid contacts were not directly quantified in this study. Therefore, lipid-associated effects should be considered a plausible explanation that needs further validation, rather than a confirmed mechanism.

At the functional level in bacteria, AME increased EtBr-associated intracellular fluorescence in MRSA. EtBr is a commonly used fluorescent substrate in efflux pump studies, and an increase in intracellular fluorescence usually indicates altered efflux-related activity. In this study, EtBr fluorescence increased rapidly after AME treatment. This result suggests that AME may affect the intracellular accumulation of EtBr or related small molecules in MRSA. It is also consistent with the computational prediction that AME may interact with the NorA binding cavity. However, EtBr is not a NorA-specific substrate. Other efflux pumps, changes in membrane permeability, altered bacterial metabolism, or disturbance of the membrane environment may also affect EtBr accumulation. Therefore, the EtBr result should be interpreted as evidence of altered efflux-associated activity or EtBr-associated intracellular accumulation. It should not be taken as direct proof of NorA-specific inhibition by AME.

The inclusion of reserpine as a pharmacological efflux inhibitor reference provided additional support for an efflux-associated interpretation of the EtBr accumulation assay. Under the same assay conditions, Reserpine increased EtBr-associated fluorescence relative to the EtBr-only control, indicating that the assay could detect pharmacologically induced changes in intracellular EtBr accumulation. AME also increased EtBr-associated fluorescence and produced a stronger and more rapid increase than reserpine under the tested conditions. This comparison supports the interpretation that AME affects intracellular accumulation of an efflux-related fluorescent substrate. Nevertheless, Reserpine serves as a pharmacological comparator rather than a NorA-specific genetic control. Therefore, the stronger AME response should not be interpreted as direct evidence of NorA-specific inhibition and may also involve effects on membrane-associated processes or other transport systems.

The ability of AME to enhance ciprofloxacin activity further supports its potential as an antibiotic adjuvant. AME alone did not show clear direct antibacterial activity at concentrations up to 512 μg/mL. When combined with AME, the MIC of ciprofloxacin decreased from 1 to 0.5 μg/mL, and the MBC decreased from 2 to 1 μg/mL. These results indicate that AME is not a potent direct anti-MRSA molecule under the tested conditions, but it can enhance ciprofloxacin-mediated growth inhibition and bacterial killing. For antibiotic adjuvants, the value of a compound does not necessarily depend on its own direct antibacterial activity, but on its ability to improve the efficacy of existing antibiotics or lower the antibiotic concentration required for bacterial inhibition. In this sense, the “weak alone but active in combination” profile of AME is consistent with its positioning as a ciprofloxacin-potentiating candidate.

The magnitude and clinical relevance of ciprofloxacin potentiation should be interpreted cautiously. In this study, AME reduced both the MIC and MBC of ciprofloxacin by twofold. This effect was measurable and reproducible under the tested conditions, but it remained modest. Importantly, this twofold shift should not be interpreted as evidence of clinical efficacy, strong synergy, or resistance reversal. Because the ciprofloxacin MIC shifted from 1 to 0.5 μg/mL in a ciprofloxacin-susceptible USA300 reference strain, the result did not represent a categorical change from ciprofloxacin resistance to susceptibility. Instead, its relevance lies mainly at the early discovery stage: AME lowered the ciprofloxacin concentration required for MRSA growth inhibition and bacterial killing *in vitro*, while showing limited direct antibacterial activity on its own. This “weak alone but active in combination” phenotype is consistent with the concept of an antibiotic adjuvant candidate. However, whether such a twofold shift can translate into a clinically meaningful effect depends on further validation in ciprofloxacin-resistant or NorA-enriched clinical isolates, intracellular ciprofloxacin accumulation assays, pharmacokinetic/pharmacodynamic considerations, and *in vivo* infection models. Because AME alone did not reach a defined MIC within the tested concentration range, the combined effect could not be strictly quantified using the standard FICI method. For these reasons, AME is more appropriately described as a natural-product-derived candidate with moderate ciprofloxacin-potentiating activity, rather than as a potent NorA inhibitor, a strong synergistic agent, or a clinically validated ciprofloxacin enhancer.

The use of a ciprofloxacin-susceptible USA300 reference strain still provides useful information for early-stage adjuvant discovery. The aim of this study was not to demonstrate reversal of ciprofloxacin resistance, but to determine whether a *F. suspensa*-derived compound could enhance ciprofloxacin activity and alter an efflux-associated fluorescence readout in a defined MRSA background. A reference strain offers a controlled starting point for candidate evaluation because it reduces the confounding effects of heterogeneous clinical resistance mechanisms, such as variable target mutations, multiple efflux systems, strain-specific permeability differences, and diverse growth phenotypes. In this context, the observation that AME had limited direct anti-MRSA activity but shifted ciprofloxacin-mediated inhibition and killing toward lower ciprofloxacin concentrations supports its preliminary adjuvant-like profile. However, the use of a ciprofloxacin-susceptible reference strain also limits the translational interpretation of the result. Therefore, this model should be viewed as a first-step screening system rather than a clinically representative resistance model. Further studies in ciprofloxacin-resistant clinical isolates, NorA-overexpressing strains, *norA*-deletion strains, and complemented strains are required to determine whether the AME-associated potentiation is retained in resistance-relevant backgrounds.

Natural products and flavonoids have been widely explored as antibiotic adjuvant candidates and efflux-modulating molecules. In previous studies, several plant-derived or natural-product-related compounds showed stronger fluoroquinolone-potentiating effects than those observed for AME in the present study. For example, capsaicin was reported to enhance ciprofloxacin activity against *S. aureus* in a NorA-associated model, with an approximately 2 log_10_ reduction in the relevant bacterial readout under the tested condition (Kalia et al., 2012). Kaempferol rhamnoside compounds were also reported to produce an approximately eight-fold reduction in the MIC of a NorA substrate antibiotic in *S. aureus* (Holler et al., 2012b). In addition, flavonoids have been systematically reviewed as bacterial efflux pump modulators, suggesting that plant-derived polyphenolic structures can serve as useful starting points for antibiotic sensitizer discovery (Waditzer and Bucar, 2021). Compared with these reported examples, AME produced a twofold reduction in ciprofloxacin MIC and MBC in the present study. Therefore, AME should be interpreted as a moderate ciprofloxacin-potentiating candidate with efflux-associated activity, rather than as a potent or target-confirmed NorA inhibitor. AME, as a biflavonoid compound, has also been reported to enhance the antibacterial activity of certain conventional antibiotics (Hwang et al., 2013). The present study links AME-mediated ciprofloxacin potentiation with EtBr accumulation and NorA-related computational prediction in MRSA.

Beyond its possible bacterial effects, the broader pharmacological profile of AME may also be relevant. AME has been reported to have anti-inflammatory, antioxidant, and macrophage-modulating activities. During infection, bacterial clearance and host inflammation are closely linked. Excessive inflammation and dysregulated macrophage responses may contribute to tissue injury and infection progression. Therefore, the potential value of AME may not be limited to direct ciprofloxacin potentiation. It may also have future relevance as a compound with both antibacterial-adjuvant and host-modulating potential. However, this study did not evaluate the effects of AME on macrophages, inflammatory cytokines, or the *in vivo* infection microenvironment. These host-directed effects should therefore be viewed as future research directions.

Overall, AME alone was not a strong direct anti-MRSA compound under the tested conditions. However, it increased EtBr-associated intracellular fluorescence and enhanced ciprofloxacin-mediated inhibition and killing of MRSA. By integrating molecular docking, MD simulation, MM/PBSA analysis, EtBr accumulation assay, MIC/MBC determination, and spot-plating validation, this study provides preliminary structural, energetic, and functional evidence for AME as a *F. suspensa*-derived ciprofloxacin-potentiating candidate.

### 4.1 Limitations

Several limitations should be acknowledged. First, the possible relationship between AME and NorA was mainly supported by molecular docking, MD simulation, MM/PBSA analysis, and EtBr accumulation assay. Direct genetic validation is still lacking. NorA-overexpressing, *norA*-deletion, and complemented strains are needed to determine whether NorA contributes to the AME-associated ciprofloxacin potentiation and EtBr accumulation phenotype.

Second, the EtBr accumulation assay cannot fully distinguish efflux-associated changes from changes in membrane permeability, bacterial metabolic state, or the involvement of other efflux systems. Although reserpine was included as a pharmacological efflux inhibitor reference in the EtBr accumulation assay, this control does not replace NorA-specific genetic validation or direct intracellular ciprofloxacin accumulation measurement. Future studies should include membrane integrity assays, membrane fluidity or lipid-order analysis, intracellular ciprofloxacin accumulation assays, and NorA-specific genetic models.

Third, this study used only the USA300 MRSA reference strain. This strain was ciprofloxacin-susceptible and was not confirmed to overexpress NorA. Although this defined reference background is useful for initial adjuvant screening and for reducing confounding from heterogeneous clinical resistance mechanisms, it cannot represent ciprofloxacin-resistant clinical MRSA populations. Therefore, the twofold MIC/MBC reduction observed in the present study should be regarded as preliminary *in vitro* evidence of ciprofloxacin potentiation, rather than as direct evidence of clinical utility or a clinically meaningful breakpoint shift. The activity of AME should be further tested in NorA-overexpressing strains, *norA*-deletion and complemented strains, ciprofloxacin-resistant strains, and clinical MRSA isolates with defined efflux profiles.

Fourth, because AME alone did not reach a defined MIC within the tested concentration range, a standard FICI value was not calculated. Therefore, the combination effect was evaluated based on ciprofloxacin MIC/MBC reduction, checkerboard viability matrix, and spot-plating validation, rather than as a strict FICI-defined synergy.

Finally, this study was mainly limited to *in vitro* assays. The cytotoxicity, pharmacokinetic properties, *in vivo* anti-infection efficacy, and potential anti-inflammatory or immunomodulatory effects of the AME-ciprofloxacin combination require further investigation in cellular and animal infection models.

## 5 Conclusion

In summary, this study identified AME as a *F. suspensa*-derived natural compound with potential ciprofloxacin-potentiating activity against MRSA. Computational analysis showed that AME could be accommodated within the NorA transmembrane cavity and maintain a relatively stable binding state in a membrane-embedded environment. MM/PBSA analysis further supported the energetic favorability of the predicted AME-NorA interaction. Functionally, AME increased EtBr-associated intracellular fluorescence in MRSA. It also reduced the MIC of ciprofloxacin from 1 to 0.5 μg/mL and the MBC from 2 to 1 μg/mL. These findings suggest that AME may serve as a *F. suspensa*-derived ciprofloxacin-potentiating candidate, possibly through effects related to efflux-associated activity.

## Data Availability

The original contributions presented in the study are included in the article/Supplementary material, further inquiries can be directed to the corresponding authors.
