# Supplementary material for: Screening of Forsythia suspensa-derived compounds identifies amentoflavone as a ciprofloxacin-potentiating candidate against MRSA
Source: Front Microbiol. 2026 Sep 3;17:1919329. doi: 10.3389/fmicb.2026.1919329 (PMC13582473; doi:10.3389/fmicb.2026.1919329)
Supplement: Supplementary file 1 [file Data_Sheet_1.pdf]

Screening of *Forsythia suspensa*-derived compounds identifies amentoflavone as a ciprofloxacin adjuvant against MRSA

Yu Fang [1*], Tianxiang Yue [1*], Jia Zhong [1], Hangsheng Zheng [1], Fanzhu Li [1#], Lai Jiang [1#]

[1] School of Pharmaceutical Sciences, Zhejiang Chinese Medical University, Hangzhou, 311140, China.

[#] Correspondence address. E-mail: lifanzhu@zcmu.edu.cn (Fanzhu Li); laijiang.sps@zcmu.edu.cn (Lai Jiang)

[*] These authors have contributed equally to this work and share first authorship.

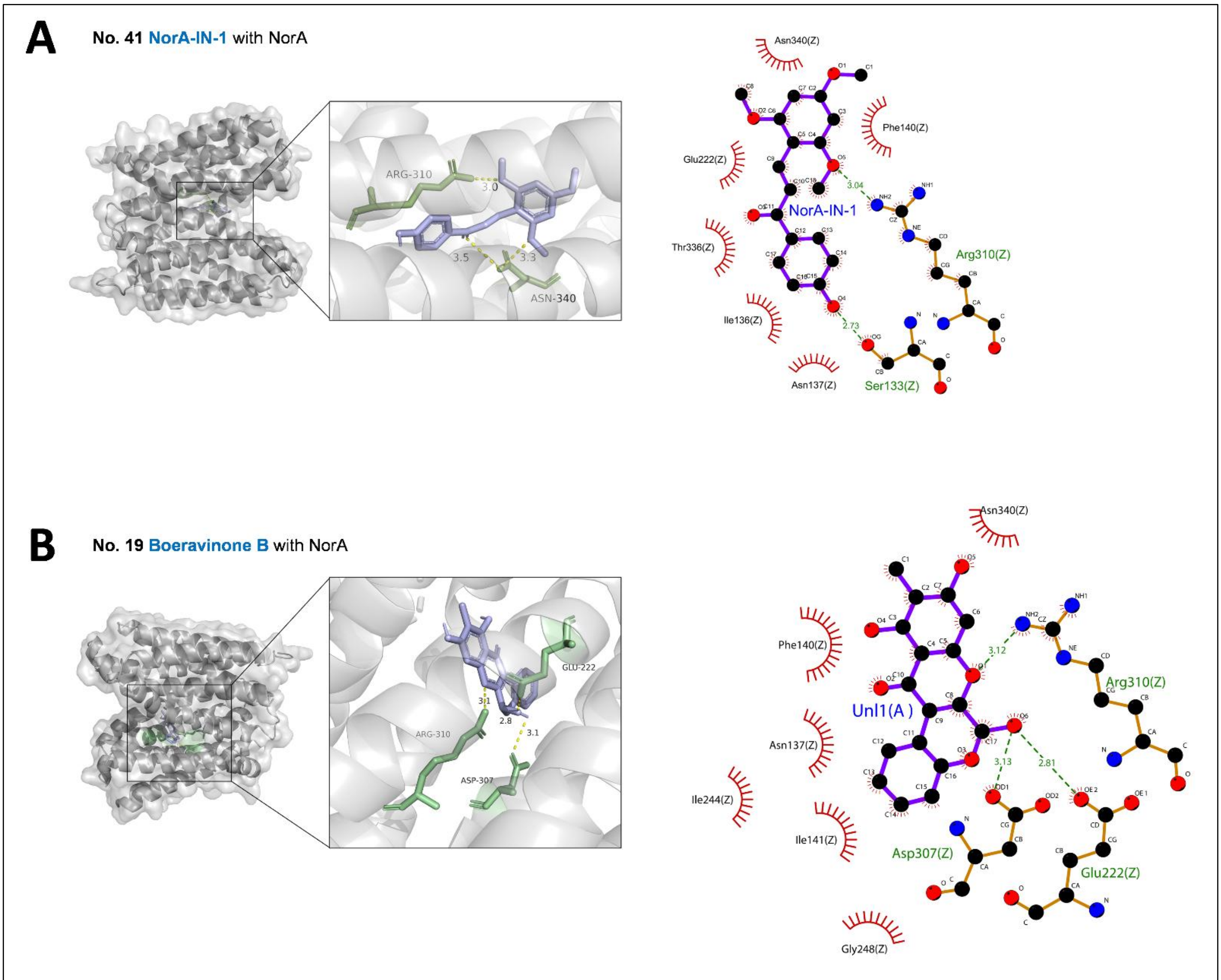

Supplementary Figure 1. Two cavities of predicted binding pose of NorA-IN-1 **(A)** and Boeravinone B **(B)** in NorA. **Left:** Three-dimensional docking model showing the predicted binding pose. Key residues surrounding the binding region are highlighted in the enlarged panel. **Right:** Two-dimensional interaction map of the NorA-IN-1-NorA complex and Boeravinone B-NorA complex, indicating multiple potential interactions within the predicted binding region.

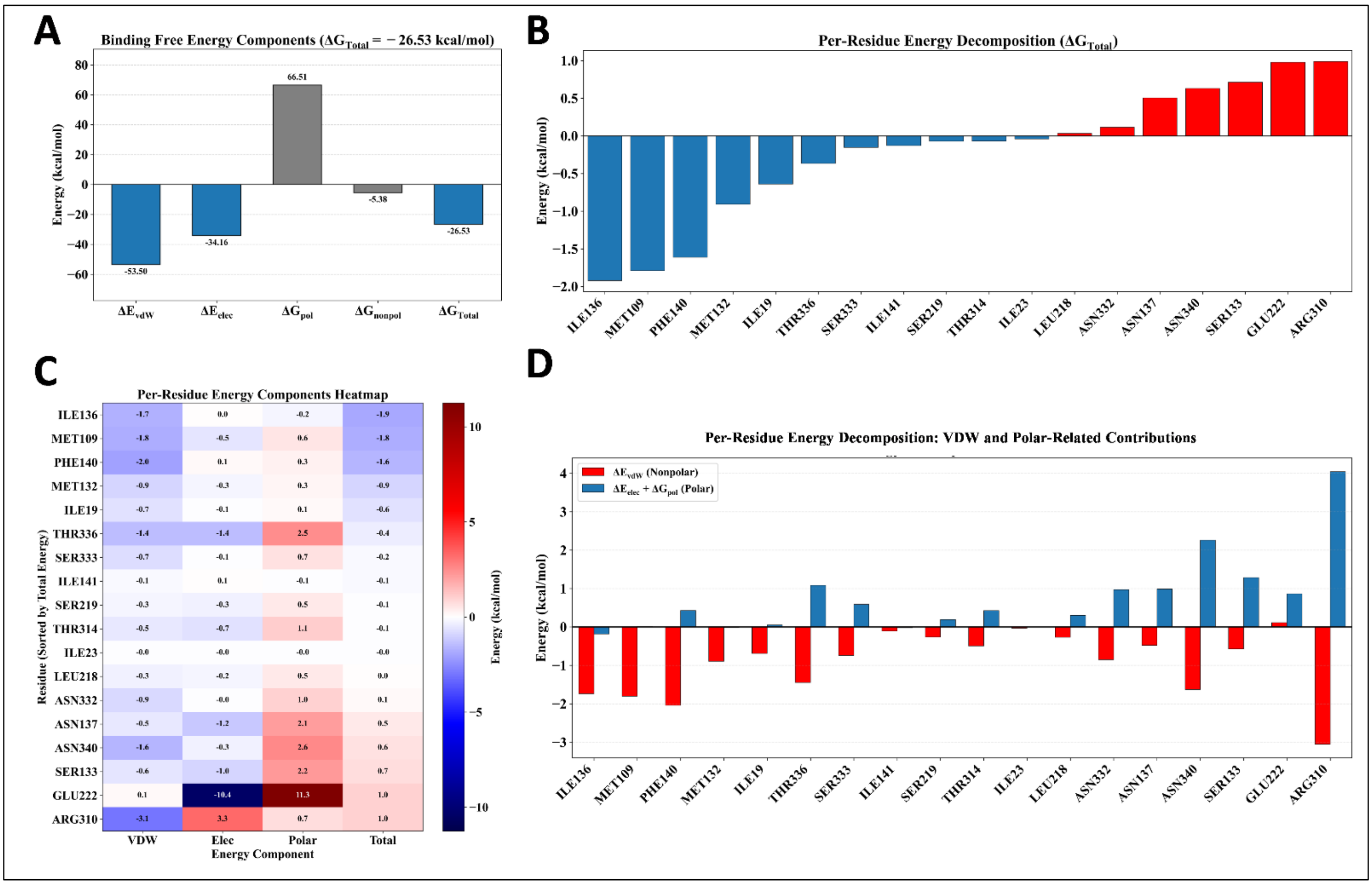

Supplementary Figure 2. MM/PBSA binding free energy analysis and per-residue energy decomposition of the AME-NorA complex. A. MM/PBSA binding free energy components of the AME-NorA complex. The estimated total binding free energy was -26.53 kcal/mol, indicating that the predicted AME-NorA interaction was energetically favorable. Van der Waals interaction contributed substantially to complex stabilization, while the polar solvation term partially offset the favorable molecular mechanics contributions. **B.** Per-residue total energy decomposition of residues surrounding the AME-binding region. Negative values indicate favorable contributions to ligand stabilization, whereas positive values indicate unfavorable or limited total energetic contributions. **C.** Heatmap showing residue-level energy components, including van der Waals, electrostatic, polar solvation, and total energy contributions. **D.** Comparison of van der Waals and polar-related contributions for selected residues. The polar-related contribution represents the sum of electrostatic interaction and polar solvation energy.

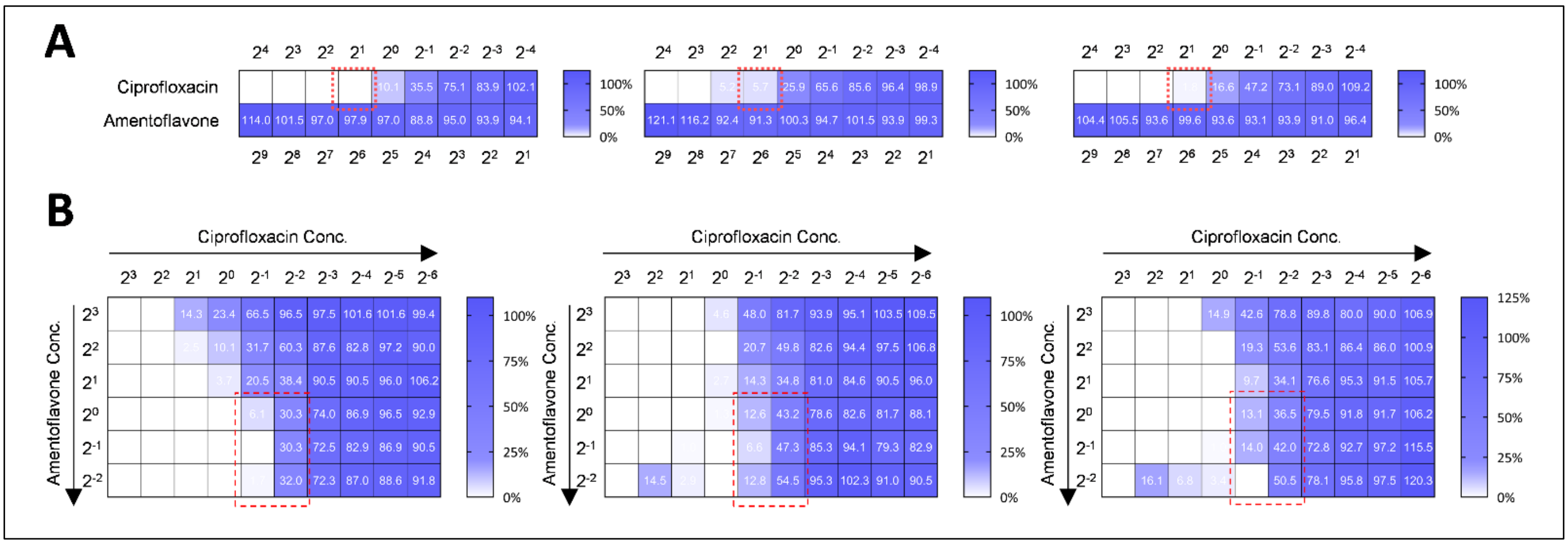

Supplementary Figure 3. Repeated viability heatmaps of ciprofloxacin, amentoflavone, and their combination against MRSA. A. Three independent repeated broth microdilution datasets corresponding to **Figure 3B**, showing the concentration-dependent effects of ciprofloxacin or amentoflavone alone. Ciprofloxacin showed clear growth inhibition within the tested concentration range, whereas amentoflavone alone did not completely inhibit MRSA growth under the same assay conditions. **B.** Three independent repeated checkerboard assay datasets corresponding to **Figure 3D**. Ciprofloxacin and amentoflavone were tested in two-fold serial dilution matrices, and bacterial viability was normalized to the untreated control. Values in each square represent relative bacterial viability (%).

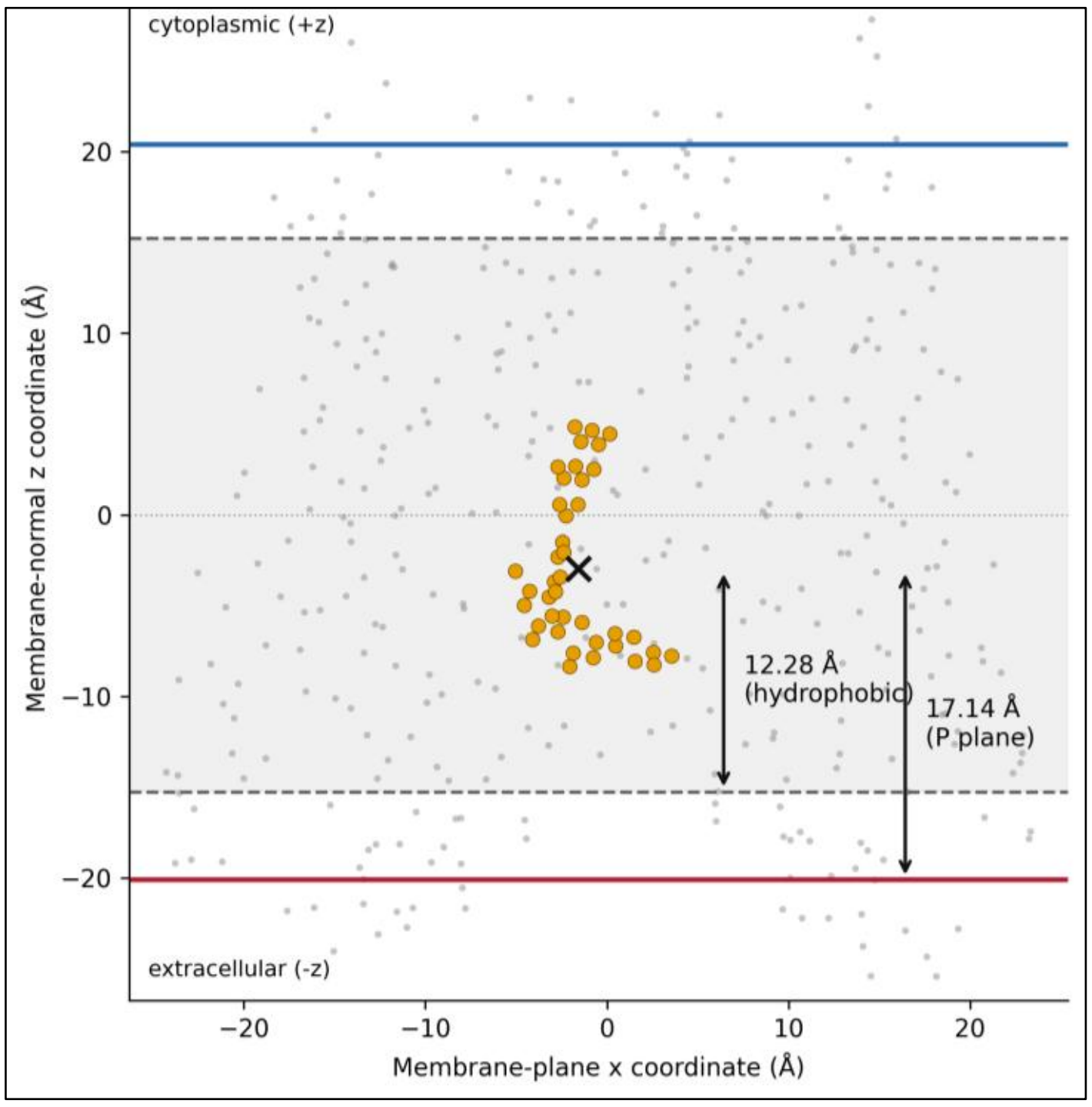

Supplementary Figure 4. Quantitative membrane-normal positions of NorA-AME. Gray dots represent protein Cα atoms, orange circles denote AME heavy atoms, and black crosses indicate the center of mass of AME heavy atoms; gray dashed lines mark the PDBTM hydrophobic boundaries, and red/blue solid lines represent the median planes of the extracellular/intracellular POPG phosphorus atoms, respectively. The scales indicate a hydrophobic interface depth of 12.28 Å and a phosphorus-plane depth of 17.14 Å.

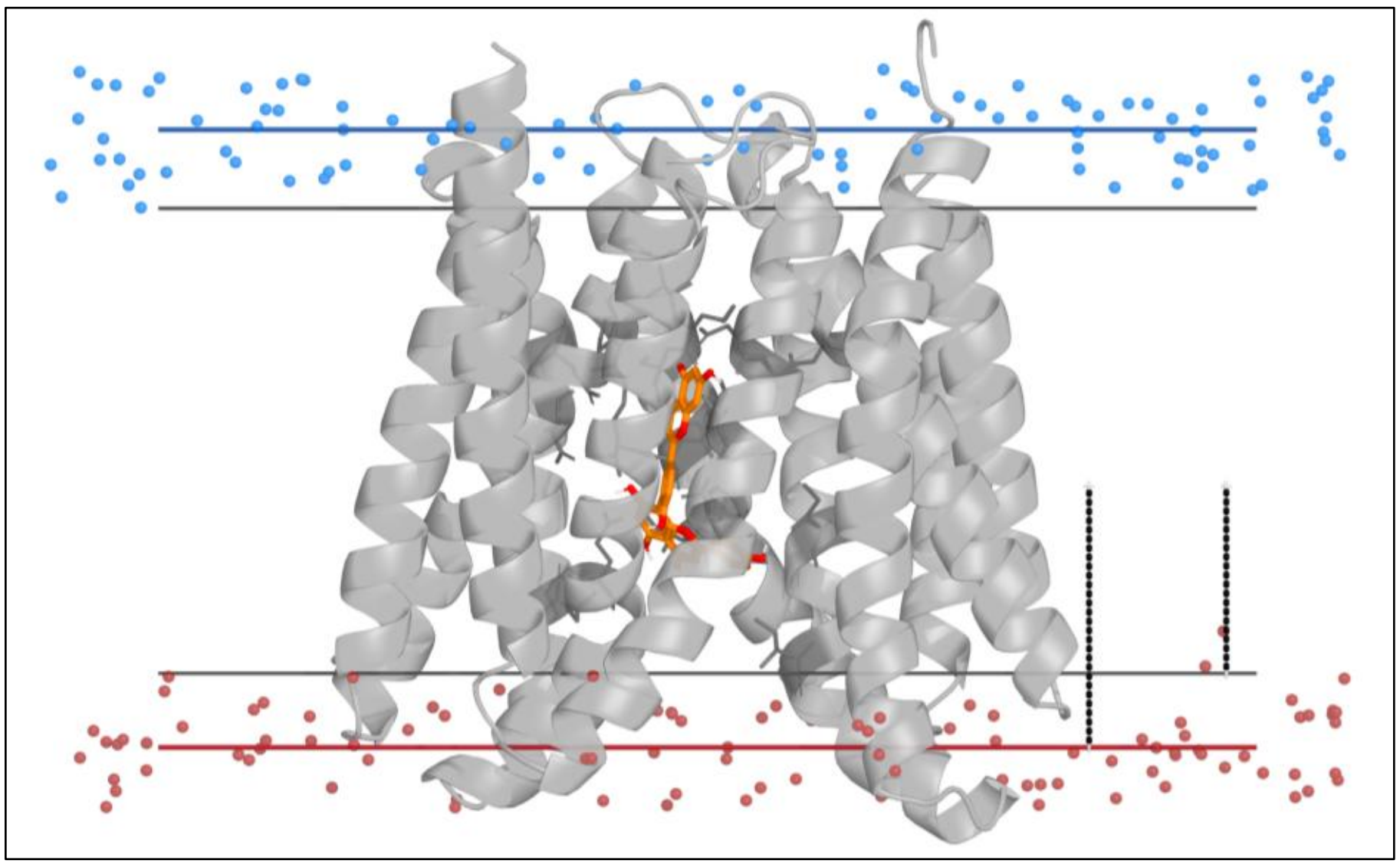

Supplementary Figure 5. Side view of the structure of NorA-AME. The protein is shown as a gray cartoon, and AME is shown as orange sticks; red/blue phosphorus atoms and solid lines represent the extracellular/intracellular POPG phosphorus planes, gray lines indicate the PDBTM hydrophobic boundaries, and the black dashed segment denotes the membrane-normal distance from the AME center of mass to the extracellular interface.

Supplementary Table 1. List of docked *Forsythia suspensa*-derived compounds and reference inhibitors with predicted NorA binding scores

TCMSP No.	Name	PubChem No.	Vina Score 1 (kcal/mol)	Vina Score 2 (kcal/mol)	Vina Score 3 (kcal/mol)	Vina Score 4 (kcal/mol)	Vina Score 5 (kcal/mol)	Average (kcal/mol)	No. in Figure 1B
1	Vanillic acid	8468	-6	-6	-6	-6	-6	-6	
2	Cymol	7463	-5.4	-5.4	-5.4	-5.4	-5.4	-5.4	
3	Citral	638011	-5.5	-5.3	-5.4	-5.7	-5	-5.38	
4	(-)-alpha-Pinene	440968	-5.2	-5.2	-5.2	-5.2	-5.2	-5.2	
5	(-)-Nopinene	440967	-5.3	-5.3	-5.3	-5.3	-5.3	-5.3	
7	Hydroxytyrosol	82755	-6	-6	-6	-6	-6	-6	
8	Wogonin	5281703	-7.4	-7.9	-8	-7.9	-7.5	-7.74	60
16	(R)-linalool	443158	-4.9	-5.4	-5	-5.3	-4.9	-5.1	
17	Safrol	5144	-6	-6.3	-6.3	-6.3	-6.2	-6.22	
18	Betulonic acid	122844	-9.4	-9.4	-9.5	-9.5	-9.3	-9.42	17
19	Myricetin	5281672	-8.3	-8.7	-8.8	-8.3	-8.3	-8.48	40
20	P-Ocimene	5281553	-5	-5.3	-5.1	-5.3	-5.1	-5.16	
21	Amentoflavone	5281600	-11.4	-11.4	-11.4	-11.4	-11.4	-11.4	14
22	Thymol	6989	-6.2	-6.2	-6.2	-6.4	-6.2	-6.24	
23	Hypericin	3663	-10.8	-10.8	-10.8	-10.8	-10.8	-10.8	31
24	Hemo-sol	440917	-5.4	-5.4	-5.4	-5.4	-5.4	-5.4	
25	()-alpha-Terpineol	442501	-6.1	-6.2	-6.1	-6.1	-6.1	-6.12	
26	Delta-Terpineol	81722	-5.9	-5.8	-5.9	-5.9	-5.9	-5.88	
27	Alpha-humulene	5281520	-7.4	-6.6	-7.4	-7.4	-6.6	-7.08	62
28	Campherenol	91747494	-6.3	-6	-6.2	-6.3	-6.4	-6.24	
31	Eugenol	3314	-5.9	-5.9	-5.9	-5.9	-5.9	-5.9	
32	Oleanolic acid	10494	-10	-9.7	-9.8	-9.9	-9.8	-9.84	43
33	(1S,5S)-1-isopropyl-4-methylenebicyclo[3.1.0]hexane	11051711	-5.5	-5.3	-5.3	-5.5	-5.3	-5.38	
34	Chebi:7	443156	-5.6	-5.6	-5.6	-5.6	-5.6	-5.6	
35	B-amyrin acetate	51398127	-10	-10	-10	-10	-10	-10	63
36	Tyrosol	10393	-5.6	-5.6	-5.6	-5.6	-5.6	-5.6	
37	Hyperin	5281643	-8.3	-8.7	-8.8	-8.7	-8.5	-8.6	32

Supplementary Table 1. List of docked *Forsythia suspensa*-derived compounds and reference inhibitors with predicted NorA binding scores (Continued)

TCMSP No.	Name	PubChem No.	Vina Score 1 (kcal/mol)	Vina Score 2 (kcal/mol)	Vina Score 3 (kcal/mol)	Vina Score 4 (kcal/mol)	Vina Score 5 (kcal/mol)	Average (kcal/mol)	No. in Figure 1B
38	[(1S)-endo]-(-)-Borneol	1201518	-5.2	-5.2	-5.2	-5.2	-5.2	-5.2	
41	(2R,3R,4S)-4-(4-hydroxy-3-methoxy-phenyl)-7-methoxy-2,3-dimethylol-tetralin-6-ol	160521	-8.3	-8.4	-8.6	-8.4	-7.6	-8.26	5
43	(2S,3R,4S,5S,6R)-2-[4-[(1R,3as,4R,6as)-4-(4-hydroxy-3-methoxyphenyl)-1,3,3a,4,6,6a-hexahydrofuro[4,3-c]furan-1-yl]-2-methoxyphenoxy]-6-(hydroxymethyl)oxane-3,4,5-triol	11168362	-9.2	-9.1	-9.2	-9.1	-9.4	-9.2	6
45	Cornoside	3084796	-7.7	-7.1	-7.2	-7.3	-7	-7.26	21
47	(3R,4R)-3,4-bis[(3,4-dimethoxyphenyl)methyl]oxolan-2-one	384877	-7.5	-8.3	-7.4	-7.7	-7.2	-7.62	7
50	Threitol	169019	-4.4	-4.5	-4	-4.4	-4.5	-4.36	
52	[(2R,3S,4S,5R,6R)-3,4,5-trihydroxy-6-[2-(1-hydroxy-4-oxo-1-cyclohexa-2,5-dienyl)ethoxy]oxan-2-yl]methyl 2-(4-hydroxyphenyl)acetate	11797786	-8.4	-9.4	-9.3	-8.5	-7.8	-8.68	8
59	Hirsutrin	5280804	-8.1	-8.6	-8.9	-8.6	-8.4	-8.52	29
61	Phillyrin	122173201	-8.8	-8.8	-8.8	-8.7	-8.9	-8.8	47
73	Mairin	64971	-9.4	-9.4	-9.4	-9.3	-9.4	-9.38	36
75	4-carboxymethylphenol	127	-6	-5.9	-5.8	-5.8	-5.8	-5.86	
76	(-)-Olivir	5273570	-7.6	-7.9	-8.3	-7.9	-7.4	-7.82	1
77	Oroxindin	3084961	-9.1	-8.9	-9.2	-8.9	-9.3	-9.08	46
82	4-hydroxy-4-[2-[(2R,3R,4S,5S,6R)-3,4,5-trihydroxy-6-(hydroxymethyl)oxan-2-yl]oxyethyl]cyclohexan-1-one	11045420	-7.4	-7.7	-7.1	-6.7	-6.9	-7.16	9
86	(-)-Phillygenin	3083590	-7.5	-7.9	-7.5	-7.7	-8	-7.72	2
87	Forsythiaside	5281773	-10.2	-10.2	-10	-9.7	-10.5	-10.12	26
89	Acteoside	5281800	-9.9	-9.8	-9.7	-9.8	-10	-9.84	11
91	Zinc00402871	6951097	-6.3	-9.9	-6.3	-6.4	-6.3	-7.04	61

Supplementary Table 1. List of docked *Forsythia suspensa*-derived compounds and reference inhibitors with predicted NorA binding scores (Continued)

TCMSP No.	Name	PubChem No.	Vina Score 1 (kcal/mol)	Vina Score 2 (kcal/mol)	Vina Score 3 (kcal/mol)	Vina Score 4 (kcal/mol)	Vina Score 5 (kcal/mol)	Average (kcal/mol)	No. in Figure 1B
95	Adoxosidic acid	13892717	-7.2	-7.4	-7.3	-7.3	-7.4	-7.32	13
97	Salidroside	159278	-7.7	-7.6	-7.7	-7.8	-7.1	-7.58	55
99	Rengyoxide	14353410	-5.2	-5.4	-5.4	-5.4	-5.2	-5.32	
102	Psuedohypericin	4978	-10	-10	-10	-10	-10	-10	51
103	Hyperforin	441298	-8.1	-8.1	-9.6	-8.1	-8.8	-8.54	30
104	Adhyperforin	9963735	-8.8	-8.6	-8.1	-9	-8.2	-8.54	12
105	Protopseudohypericin	171335	-10	-9.9	-10	-9.9	-10	-9.96	50
106	Protohypericin	164660	-10.5	-10.5	-10.4	-10.5	-10.4	-10.46	49
108	Procyanidin	107876	-10.1	-9.9	-9.5	-11.3	-10.9	-10.34	48
109	Emodinanthrone	122635	-8.4	-8.5	-8.4	-8.4	-8.4	-8.42	23
110	(2R,3R,4R)-2-(3,4-dihydroxyphenyl)chroman-3,4,5,7-tetrol	11208959	-7.6	-7.6	-7.6	-7.8	-7.7	-7.66	4
111	Olivil	44566587	-7.4	-7.5	-7.6	-7.5	-7.5	-7.5	44
113	(1R,3ar,4R,6as)-1,4-bis(4-hydroxy-3-methoxyphenyl)-3,3a,4,6-tetrahydro-1H-furo[3,4-c]furan-6a-ol	21632947	-7.8	-7.6	-7.6	-7.8	-7.6	-7.68	3
114	Euxanthone	5281631	-7.9	-7.9	-7.9	-7.9	-7.9	-7.9	24
115	Myrcenol	10975	-5	-4.7	-4.8	-4.9	-4.9	-4.86	
116	Norlapachol	231114	-7.4	-7.4	-7.4	-7.3	-7.4	-7.38	42
117	(1S,6R)-3,7,7-trimethylbicyclo[4.1.0]hept-3-ene-2,5-dione	10057860	-6.1	-6.1	-6.1	-6.1	-6.1	-6.1	
123	Myricadiol	3084282	-10.1	-10.2	-10.3	-10.1	-10	-10.14	39
126	Onjixanthone I	5320290	-7	-7	-7	-6.9	-7.1	-7	45
127	Safynol	5281155	-6.3	-6.3	-6.2	-6.3	-6.2	-6.26	
128	Scopine	1274465	-5.2	-5.1	-5.2	-5.2	-5.2	-5.18	

Supplementary Table 1. List of docked *Forsythia suspensa*-derived compounds and reference inhibitors with predicted NorA binding scores (Continued)

TCMSP No.	Name	PubChem No.	Vina Score 1 (kcal/mol)	Vina Score 2 (kcal/mol)	Vina Score 3 (kcal/mol)	Vina Score 4 (kcal/mol)	Vina Score 5 (kcal/mol)	Average (kcal/mol)	No. in Figure 1B
130	Urushiol III	5281862	-6.9	-7.5	-7.4	-7.7	-7.7	-7.44	59
131	Succinic acid	21952380	-4.8	-4.6	-4.8	-4.8	-4.6	-4.72	
132	Sitogluside	5742590	-10.3	-10.5	-10.2	-10.2	-10.2	-10.28	56
133	Beta-sitosterol	222284	-9.2	-9.5	-9.7	-9.6	-10.1	-9.62	64
134	Caffeate	1549111	-6.4	-6.4	-6.4	-6.4	-6.4	-6.4	
135	Rutin	5280805	-9.3	-9.6	-9.3	-9.5	-9.7	-9.48	54
136	Kaempferol	5280863	-7.7	-7.7	-7.8	-7.8	-7.7	-7.74	33
137	Ursolic acid	64945	-10.1	-10	-10.1	-10.2	-10	-10.08	58
138	Arctiin	100528	-9	-9	-8.6	-8.1	-8.8	-8.7	16
139	Arctigenin	64981	-7.4	-7.6	-7.8	-7.8	-7.2	-7.56	15
140	Matairesinoside	486612	-8.1	-8.6	-8.6	-9	-8.7	-8.6	38
142	Luteolin	5280445	-8.4	-8.3	-8.4	-8.3	-8.3	-8.34	35
143	Palmitic acid	985	-5.9	-5.2	-5.6	-5.2	-5.2	-5.42	
144	B-terpineol	8748	-5.4	-5.3	-5.2	-5.4	-5.4	-5.34	
145	Guajavarin	5481224	-8.9	-8.5	-9.1	-8.5	-8.5	-8.7	28
146	Bicuculline	10237	-8.8	-9	-9	-9	-9.1	-8.98	18
147	Stearic acid	5281	-5	-5.2	-5.6	-5	-5.1	-5.18	
148	Terpilene	7462	-5.4	-5.4	-5.4	-5.3	-5.3	-5.36	
149	Linalool (d)	67179	-4.8	-5.4	-5.2	-5.4	-5.2	-5.2	
150	Quercetin	5280343	-8	-8.2	-7.9	-8.1	-8.2	-8.08	53
151	Suspensaside A	132550846	-10.7	-10.2	-10.2	-10.3	-10.1	-10.3	57
155	Pubchem ID: 163011695	163011695	-9.4	-10.1	-9.6	-10.2	-9.8	-9.82	52
160	Matatabiether	5319300	-5.9	-5.8	-5.9	-5.9	-5.8	-5.86	
161	Matairesinol	119205	-8.1	-8.2	-8.3	-8	-7.6	-8.04	37
162	Lactucasterol	5318918	-9.7	-9.8	-9.8	-9.9	-9.6	-9.76	34
165	Forsythinol	363707	-5.5	-5.7	-5.5	-5.6	-5.1	-5.48	
166	Forsythide	15559328	-7.5	-7.6	-7.5	-7.8	-7.8	-7.64	27
167	Forsythialan B	44453332	-8	-7.9	-7.5	-8	-7.4	-7.76	25

Supplementary Table 1. List of docked *Forsythia suspensa*-derived compounds and reference inhibitors with predicted NorA binding scores (Continued)

TCMSP No.	Name	PubChem No.	Vina Score 1 (kcal/mol)	Vina Score 2 (kcal/mol)	Vina Score 3 (kcal/mol)	Vina Score 4 (kcal/mol)	Vina Score 5 (kcal/mol)	Average (kcal/mol)	No. in Figure 1B
171	Egenine	189685	-8.6	-8.5	-8.7	-9.5	-8.5	-8.76	22
172	Darwinol	636475	-5.4	-5.4	-5.4	-5.4	-5.5	-5.42	
173	Cleroindicin B	184824	-5.7	-5.8	-5.1	-5.6	-5.6	-5.56	
174	Cedrusin	11210164	-7.8	-7.7	-7.8	-7.8	-7.7	-7.76	20
175	Camphor	159055	-5.2	-5.2	-5.2	-5.2	-5.2	-5.2	
177	8-hydroxypinoresinol	3010930	-8	-8.3	-8.2	-7.3	-8.2	-8	10
Ref 1	Nor-IN-1	10495447	-7.3	-7.4	-7.4	-7.4	-7.5	-7.4	41
Ref 2	Boeravinone B	14018348	-8.7	-8.7	-8.8	-8.8	-8.8	-8.76	19

Supplementary Table 2. MRSA viability of ciprofloxacin, and the combination of ciprofloxacin & AME.

	**Cip Conc. (4 µg/mL)**	**Cip Conc. (2 µg/mL)**	**Cip Conc. (1 µg/mL)**
**Viability % (Mean±SD)**	-4.22±8.21 %	0.00±6.75 %	17.57±7.92 %
		**Cip Conc. (1 µg/mL)**	**Cip Conc. (0.5 µg/mL)**
**Viability % (Mean±SD)**	**AME Conc. (1 µg/mL)**	-1.54±2.48 %	10.63±3.92 %
	**AME Conc. (0.5 µg/mL)**	-4.80±6.11 %	6.57±7.46 %
	**AME Conc. (0.25 µg/mL)**	0.00±3.34 %	3.94±7.96 %

Data are derived from **Supplementary Figure 3** and are presented as mean ± SD. Bacterial viability values were normalized to the untreated control. Slight negative values reflect background correction and were interpreted as near-complete growth inhibition. MICs were determined based on broth microdilution/visual growth assessment together with the viability heatmap, whereas MBCs were determined by spot-plating results showing no visible CFU.

Supplementary Table 3. Specific data of the membrane reference planes.

**Reference plane**	**Z median (Å)**	**Interquartile range (Å)**	**MAD (Å)**
Extracellular POPG phosphorus plane	−20.108	−21.812 to −18.077	1.953
Intracellular POPG phosphorus plane	+20.407	+18.421 to +22.109	1.903
Extracellular PDBTM hydrophobic boundary	−15.250	fixed reference plane	N/A
Intracellular PDBTM hydrophobic boundary	+15.250	fixed reference plane	N/A

Supplementary Table 4. Depth metrics of NorA-AME

**Metric**	**Value**	**Definition / Explanation**
AME heavy-atom centroid z	−2.966 Å	Relative to the membrane midplane, slightly biased toward the extracellular side
Distance to extracellular hydrophobic interface	12.284 Å	Primary reported value
Distance to extracellular POPG phosphorus plane	17.142 Å	Complementary value for the headgroup plane
Extracellular phosphorus‑plane MAD	1.953 Å	Static headgroup distribution width
5‑Å contact residues	20	Number of pocket‑lining residues

Supplementary Table 5. Molecular span and pocket location

**Metric**	**Range or value**
AME distance to extracellular hydrophobic interface	6.912–20.098 Å
AME distance to extracellular POPG phosphorus plane	11.770–24.956 Å
Proportion of AME heavy atoms within the hydrophobic layer	100%
Depth of pocket‑lining protein atom centroid	15.161 Å (to hydrophobic interface);<br>20.019 Å (to phosphorus plane)
